# Asylum Seekers and Swiss Nationals with Low-Acuity Complaints: Disparities in the Perceived level of Urgency, Health Literacy and Ability to Communicate—A Cross-Sectional Survey at a Tertiary Emergency Department

**DOI:** 10.3390/ijerph17082769

**Published:** 2020-04-17

**Authors:** Karsten Klingberg, Adrian Stoller, Martin Müller, Sabrina Jegerlehner, Adam D. Brown, Aristomenis Exadaktylos, Anne Jachmann, David Srivastava

**Affiliations:** 1 Emergency Department, University Hospital Bern, 3010 Bern, Switzerland; karsten.klingberg@insel.ch (K.K.); neocortex81@gmail.com (A.S.); martin.mueller2@insel.ch (M.M.); sabrina.jegerlehner@insel.ch (S.J.); aristomenis.exadaktylos@insel.ch (A.E.); anne.jachmann@insel.ch (A.J.); 2 Accident & Emergency, Barts Health NHS Trust, London EC1A 7BE, UK; 3 Department of Psychology, New School of Social Research, New York, NY 10011, USA; brownad@newschool.edu

**Keywords:** health-seeking behavior, access to health care, emergency department, refugee, asylum seeker, non-urgent complaints, migrants

## Abstract

*Background:* Emergency departments (EDs) are being increasingly used for low-acuity conditions and as primary care providers. Research indicates that patients with the status of asylum seeker (AS) may be seeking care in EDs at higher levels than nationals. The aim of this study was to identify disparities in the use of emergency care between AS and Swiss nationals (SN) with non-urgent complaints. *Methods:* Data were obtained from a survey in the period 01/12/2016–31/07/2017 of walk-in low-acuity patients attending the ED of the University Hospital Bern (Switzerland). AS and a gender, age-matched control group of SN of ≥16 years of age were included. Sociodemographic and survey data comprised information about health-seeking behavior in the home and reception country, knowledge of health care systems (HCSs), barriers to care and perceived acuity of the visit. Furthermore, attending physicians assessed the level of urgency of each case. *Results:* Among AS patients, 30.2% reported that they had no knowledge of the Swiss HCS. In total, 14.2% considered that their medical needs were non-urgent. On the other hand, 43.4% of the attending physicians in the ER considered that the medical needs were non-urgent. This contrast was less pronounced in SN patients. The majority of AS (63.2%) and SN (67.6%) patients sought care from the ED without first contacting a GP. In 53.8% of cases, an interpreter was needed during the ED consultation. *Conclusions:* Several factors associated with health-seeking behavior in the ED differed between AS and SN patients. Measures to increase health literacy, provision of easily accessible primary care services and intercultural-trained staff could improve quality of care and reduce the usage of EDs as primary care providers.

## 1. Introduction

In recent years, there has been an unprecedented increase in the numbers of individuals experiencing forced migration, with many seeking refuge in countries throughout Europe [1]. In particular, Switzerland has witnessed a sharp rise in persons seeking asylum [2]. The net population growth from AS and refugees in countries such as Switzerland has important public health implications since there will be a greater health care demand [3]. Research on hospital-based emergency department (ED) utilization in Norway has shown that immigrants use emergency health care services significantly more often than nationals [4]. These findings are consistent with other studies in Europe that show that recent immigrants are more likely than local nationals to seek care from EDs and out-of-hours GP services [5,6,7,8]. During the asylum process in Switzerland, every individual is granted with universal health care coverage and a GP-based model of care, which gives the asylum seekers access to a GP including regular consultations in the asylum center. Additional ED visits are possible without prior GP consultation and there are no co-payments necessary.

General trends showing that hospital-based EDs face increasing levels of visits throughout the world [9,10,11,12,13]. This means that EDs are confronting growing pressure to meet the needs of patients with insufficient resources, resulting in a variety of “supply” problems such as overcrowding, boarding, higher morbidity, and staff burn out [9,14].

Patients seeking care for non-acute medical issues appear to make up a large percentage of ED visits, ranging up to 62%, with a mean of 37% [15]. Triage data from a study of North African patients who had recently migrated to Switzerland showed that they were less likely to need highly urgent care [16]. In a recent interview-based study of low-acuity ED patients in Germany, two factors were identified [17]: firstly, patients felt it would be more convenient to present in the ED, as this did not require an appointment and was not restricted to office hours. Secondly, patients believed they would receive more specialized advice. In addition, poor health and socioeconomic status have been shown to be important factors that influence the threshold of ED use for non-urgent complaints [18]. These findings are supported by Kraaijvanger et al., who showed that health concerns, access to medical tests (e.g., X-rays, blood tests, etc.) and convenience are strongly associated with ED visits for non-acute issues [19].

Identifying the underlying factors contributing to consultations for non-urgent complaints could guide stakeholders and policy makers in implementing measures for equal and effective health care—especially for this vulnerable population. To understand and manage the influencing factors that force AS to seek help in the ED for non-urgent complaints will help to improve services and quality of care for those who may be unable to navigate in a new health care system and have to use the ED as an entry point to the health care system. Therefore, the purpose of this study was to conduct interviews with AS and SN patients, in order to understand the different factors that influence consultations in the ED for non-urgent conditions. 

## 2. Materials and Methods

### 2.1. Study Design, Setting and Participants

We conducted a prospective cross-sectional, controlled, single-center study. Data were collected from 01/12/2016 to 31/07/2017 among patients attending the ED of the University Hospital Bern (Inselspital) in Switzerland. The Inselspital is one of the largest hospitals in Switzerland, with a catchment area of 1.8 million people. More than 45,000 patients are treated in the ED each year [20]. Eligible AS were matched for a predefined period with a group of SN as controls. The two groups were matched by sex, age and triage category. The STROBE (STrengthening the Reporting of OBservational studies in Epidemiology) guideline for cross-sectional studies was employed [21].

Walk-in AS patients attending the ED during the study period and of 16 years of age or older were asked to participate in the survey. Their asylum status was defined by the official Swiss identification card (“F”: provisionally admitted foreigners, “N”: permit for asylum seekers, or “S” people in need of protection). The study was restricted to patients who had no life-threatening or highly urgent problem as defined by the category in the Swiss Emergency Triage Scale (STS) (Range from 1: acute life-threating to 5: non-urgent problem [22]).

The criteria for eligibility in the SN control group included registration with Swiss citizenship, together with the same triage category, age +/−10 years as well as same sex as the matching AS. Efforts were made to obtain a close temporal match, e.g., recruiting a successive control patient shortly after successfully recruiting an AS study patient. The control group was recruited in a predefined, reduced period. Exclusion criteria for both groups were critically ill patients by the STS (STS < 3), the need for expedited diagnostic testing as judged by the attending consultant, transport by ambulance or patient’s refusal to participate in the study. 

We designed a survey with questions regarding prior and current health-seeking behavior based on current literature and with the help of a psychologist (YB). The survey was available both as a printed and as a protected web-based version on a tablet. 

Trained medical students acted as interviewers and conducted the survey in the waiting area or the treatment area without interfering with medical care. The students received an allowance per questionnaire but were not involved in the treatment.

In cases, where communication between the participant and the interviewer was not possible due to language barriers, accompanying persons or professional interpreters were consulted by phone or in person, to ensure that the participant understood the consent form and the survey questions. These interpreters then also facilitated the medical care (not part of the study).

### 2.2. Measures

The survey consisted of a nineteen-item patient questionnaire and a nine-item physician questionnaire. The patient questionnaire covered questions about the patient’s demographics and socioeconomic status (SES), including education, language skills and current employment situation. Furthermore, all participants were asked about their health-seeking behavior, including previous visits to a general practitioner (GP), the perceived level of urgency of the current visit, their motives in seeking help in the ED and their knowledge of the Swiss health care system (HCS). The attending physician assessed the urgency of the consultation and the discharge status.

### 2.3. Statistical Analysis

The distribution of categorical variables is given with the absolute number and the relative number as a percentage. The distribution of continuous variables, such as age or length of stay, is described as medians with interquartile (IQR) ranges. Unless otherwise stated, the Chi-squared test was used to test for significant differences between the study and control groups for categorical variables and the Mann–Whitney U test was used for continuous variables, as they were not normally distributed. Data were entered using Microsoft Office Excel 2016 for Windows 10 (Version 1805, Microsoft Corporation, Redmond, WA, United States). All statistical analyses were performed with IBM SPSS Statistics Version 25 (Armonk, New York, NY, United States). Statistical significance was considered at a *p* value smaller than 0.05. Graphs were created using Microsoft Office Excel 2016 for Windows 10 (Version 1805, Microsoft Corporation, Redmond, WA, United States).

### 2.4. Compliance with Ethical Standards

Study participation was voluntary, free of any compensation and individual verbal and written patient consent was obtained before answering the survey. Patient-related information was anonymized prior to analysis. The study was presented to and approved by the regional ethics committee of the Canton of Bern, Switzerland (06.10.2016, KEK-BE: 2016-01662). The study—including data collection and extraction, anonymization, analysis, and storage—was performed in accordance with Swiss law, the standards of the local ethics committee and the Declaration of Helsinki [23].

## 3. Results

### 3.1. Demographics

In total, 557 AS patients were admitted to the ED during the study period. In total, 168 patients were excluded because of admission by ambulance services. Another 38 were excluded because of a higher triage category (STS 1 or 2). In total, 351 AS met the inclusion criteria. In total, 237 patients of the eligible collective could not be included due to circumstances in the ED, e.g., study team unavailable. The analysis excluded four individuals who refused consent and four patients who, after giving consent, did not answer the questions during the interview for unknown reasons. Questionnaires with partially missing answers were included in the study. Missing answers are marked accordingly in the results section. A total of 106 AS were included in the analysis. The control study group of 68 SN was subsequently recruited during a restricted period—after matching for age (+/−10 years), gender and STS. 

The demographic characteristics of the groups are summarized in Table 1. The AS patients were predominantly from two geographical regions (cumulatively 70.8%), with Eastern Africa accounting for 40.6% and Western Asia for 30.2% of cases, followed by Southern Asia for 17%, Northern Africa for 5.7% and Western Africa for 4.7%. Only one individual originated from Southern Europe. More than half of the AS were unemployed and nearly two-thirds of the patients were males. There was a significant difference (*p* < 0.001) between the groups in work status (unemployed vs. employed/self-employed vs. other (student/retired/housewife/man)). 

**Figure 1:**
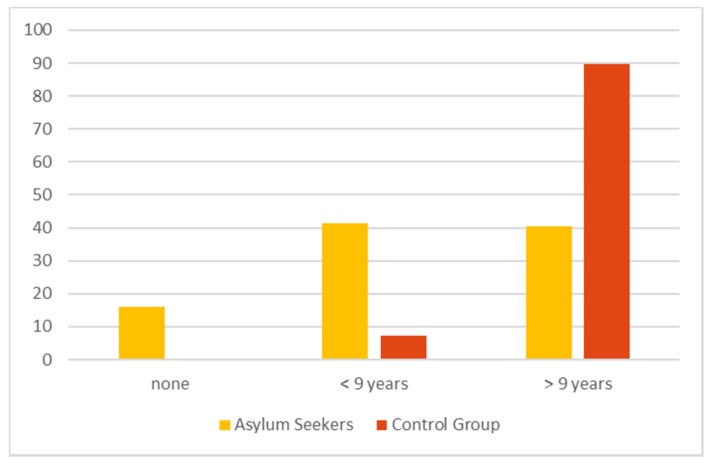
Formal education (years in school), numbers in % (missing answers: asylum seeker (AS) 2, control group (CG) 2).

The level of education (see Figure 1) was less than 9 years in 41.5% of the AS and 16% reported that they had no formal education at all. The level of the reported formal education was significantly lower in the AS group than in the control group (*p* < 0.001).

### 3.2. Health Care Knowledge

Almost one-third of the AS (*n* = 32/106; 30.2%) reported that they had had no knowledge of the Swiss HCS, with the greatest unawareness being within 3 months of arrival. Within this group, only four persons (*n* = 4/13; 30.8%) reported that they had received information about the HCS at the reception center. This number rose to 28 persons (*n* = 28/55; 50.9%) among those with a length of stay of between three months and two years.

With increasing length of stay in Switzerland, AS patients reported that they had acquired health care knowledge from family and friends. In contrast, knowledge acquired using the provided media (e.g., printed brochures, general advertisement, internet-based information sources) was very low—at 4.7% overall. After more than 2 years in Switzerland, 11 persons (*n* = 11/38; 28.9%) still reported no knowledge of the health care system. This was compared to 7.4% of the SN control group.

**Figure 2:**
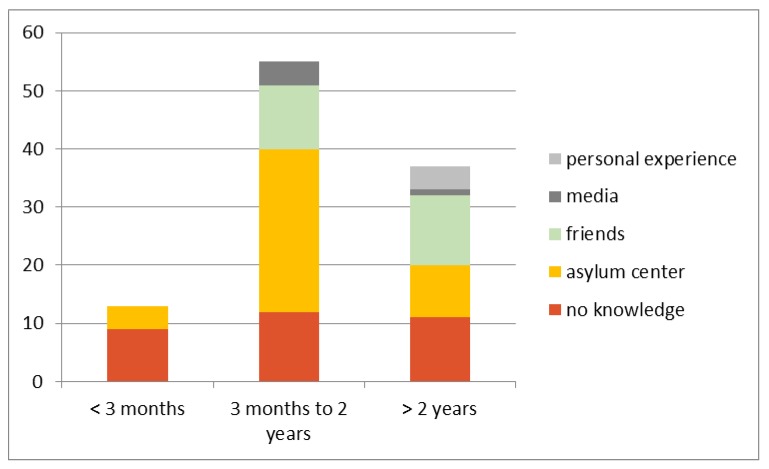
Health literacy of asylum seekers and source of knowledge of the Swiss health care system (HCS), dependent on the length of stay in Switzerland, total numbers (missing answers: 1).

Figure 2 shows an overview of the knowledge and source of information regarding the Swiss HCS within the AS group. 

### 3.3. Health Care Utilization

**Figure 3:**
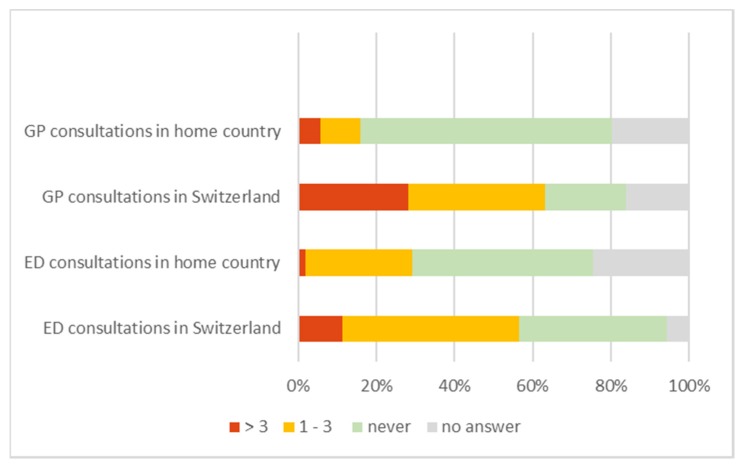
Chart of general practitioner (GP) and emergency department (ED) consultations of AS in their home country and in Switzerland, numbers in %.

In total, 26.4% of the group of AS patients had a GP in their country of origin, which increased to 67.9% in the reception country. Occasional use of the ED in their pre-migration countries (1–3 times a year) was reported by 27.4% of AS patients versus 35.3% of SN patients. In general, there is a significant increase in the usage of primary care services in Switzerland (McNemar Test for GP usage in home country vs. Switzerland (*p* = 0.001) and ED usage (*p* = 0.029)), even though the use of EDs was already more pronounced than GP use in their home countries. However, a high rate of missing responses to these questions warrants special care when interpreting these results. The comparison between the utilization of health care within the pre-migration country and Switzerland is displayed in Figure 3.

### 3.4. Barriers to Care

Communication between the AS patient and the physician without an interpreter was possible in 45.3% of cases. More than half (53.8%) of the AS patient group were not able to communicate directly with the physician, as they did not speak a national language (e.g., German, French, Italian) or English. In 70.2% of these cases, accompanying persons, such as family and friends, acted as interpreters.

Approximately one-third (35.8%) of the AS patients had tried to consult the physician in their asylum center (15.1%) or their attending GP (20.8%) before consulting the ED. Nevertheless, the majority of AS patients (63.2%) and of the control group (67.6%) sought care from the ED without first contacting a GP or a physician from their asylum center. The reasons given for these decisions are shown in Table 2. The most common response (29.9%) by the AS patients was not having a GP, followed by consultations outside GP opening hours (25.4%). In total, 19.4% of the AS stated previous poor experiences with GP services or expected better care at the ED. In contrast, SN only half as often (13%) reported that they had no GP and the most common reason given (32.6%) was that they had directly consulted the ED because their visit was outside GP opening hours.

There were no significant differences (*p* = 0.223) between AS patients and SN with respect to seeking care during working hours (08:00–18:00, weekdays Monday–Friday). It is striking that more than half of the consultations in both AS and SN patients took place within general working hours. The length of stay in the ED did not differ significantly between the two groups (*p* = 0.141)—the median LOS of the SN control group was 3:22 h (IQR: 2:40–5:25 h) and the median LOS of the AS group was 3:09 h (IQR: 2:01–5:00 h)—neither did the respective proportions of patients receiving inpatient versus outpatient treatment (*p* = 0.892).

### 3.5. Patient Perceptions of Medical Urgency

Over one-third (34.9%) of the AS patients perceived a need for treatment within one hour, whereas the attending physician assigned this level of urgency to only 11.3% of cases. In addition, 14.2% of the enrolled AS patients regarded their problem as non-urgent and this assessment was shared by the attending ED physician in 43.4% of these cases.

**Figure 4:**
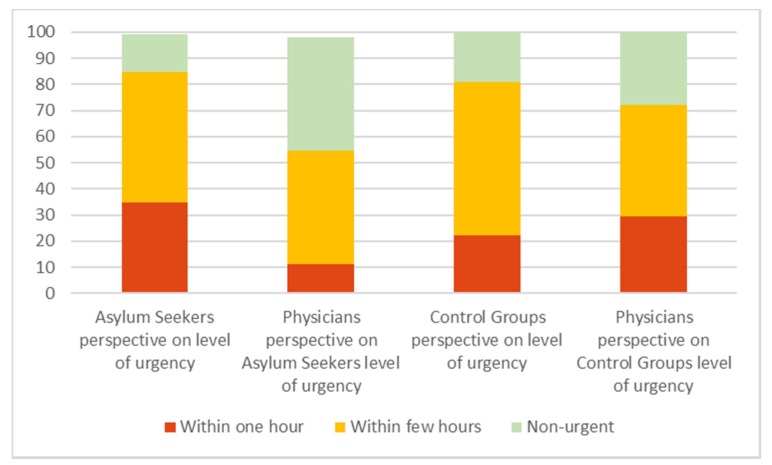
Assessment of the estimated level of urgency by patient vs. physician; number in % (missing answers: AS 3, CG 0).

In total, 22.1% of the SN patients perceived a need for treatment within one hour, whereas the attending physician assigned this level of urgency to 29.4% of cases. In addition, 19.1% of the enrolled SN patients regarded their problem as non-urgent and this assessment was shared by the attending ED physician in 27.9% of these cases. The estimated level of urgency from the patient’s perspective compared to the physician’s perspective is displayed in Figure 4.

The subjective level of urgency by patients and physicians did not influence the initially allocated triage level. All patients included in this study were categorized in STS 3–5 prior to consultation and the item “subjective level” was used only to display the perceived level of urgency.

## 4. Discussion

AS and SN differed in their reasons for seeking care in the ED, their knowledge of the Swiss HCS, and their perceptions of medical urgency. However, other factors, such as length of stay, discharge type, and time of visit did not differ between the two groups.

Although we are unable to determine from this study whether these findings are representative of AS patients generally, the differences observed between the AS and SN patients may shed light on the observed high usage of the ED by AS patients in recent years (e.g., Müller et al. 2016 [3]). These findings suggest that the factors associated with seeking care in the ED among AS patients are multifactorial and reflect potential gaps in health care knowledge, linguistic barriers, and perceptions of acuity and care. The differences in health care utilization, represented by the use of PC and ED care in the home country versus Switzerland, might not be due to differences in the individual health-seeking behavior but represent effects of the different national health care systems and the availability of primary and emergency care. 

Attempts to help guide and inform health-seeking behavior by AS patients will benefit from improvements in a range of social and cultural factors that influence the dissemination of health care information, coupled with training for medical staff working with the AS community.

Firstly, AS patients’ lower levels of knowledge of the Swiss HCS could be due to a lack of education and general health care experience, as the usage of primary care services in their home countries was significantly lower than in Switzerland. Furthermore, information about health care appears to be gradually acquired through informal networks such as friends and family members. Such findings suggest that policy makers and those working in health care promotion may be able to reduce non-urgent visits through public health campaigns such as peer group interventions [24].

A second key factor contributing to ED use among AS patients may be actual or anticipated poor care from GPs. AS patients were more likely to say that they had had a poor experience with a GP or expected to receive better care in the ED. These findings warrant further investigation, as the specific nature of the poor experiences and the perceptions around quality of care cannot be determined from this study. A combination of public health campaigns and culturally informed training for GPs may help to improve perceptions and actual experiences of care during GP visits [25].

A third difference between AS and SN patients was around perceptions of urgency. Even though the majority of both groups sought care in the ED without prior consultation by a GP, the perceived level of urgency among SN was closer to the assessment of the attending physicians. One reason that AS patients may be more likely to seek care in the ED is that they believe their symptoms are of higher acuity. Education of AS patients on how to identify acute symptoms and better access to primary care for non-urgent complaints may help to improve quality of care [26].

More generally, these findings provide further information about the major gaps in interpretation in health care contexts for AS patients. As these data show, a majority of AS patients are not able to communicate easily with their medical providers, and often rely on family members and even children to broker these interactions. The reliance on untrained interpreters may be associated with miscommunication, misdiagnosis, and poor outcomes and includes safety concerns in relation with human trafficking [27,28,29].

The level of education reported by the AS group was significantly lower than in the SN group. Furthermore, the work status differed significantly, as most AS patients were unemployed, but most SN were employed or self-employed. Low socioeconomic status is linked to the overuse of ED care [18]. This result was reproduced here but we did not investigate the nature of the association. 

The differences identified between the AS and SN may help to address the increase in non-urgent visits to hospital-based EDs [3,5]. The findings suggest several different approaches to improving the access of this vulnerable population to adequate and equal care. 

## 5. Limitations

This survey was restricted to AS and SN patients with non-urgent problems and in the context of an urban university hospital in a single-center setting. There are therefore several different selection biases. Furthermore, this study does not cover an entire year, so there might be the risk of seasonality in data collection. Additionally, there were a number of randomly missing answers, which limits the interpretation of the results. The strong skew towards young males in the study group proved to be hard to match in the control group. A large peak at the outmost higher end of the age group admitted to the adult ED department was compounded by the permitted age variation of +/−10 years. The average age of ED patients in Switzerland is above 50 years [30]. One limitation is the self-reported answers, which may be positively or negatively biased, according to the participant’s impression that answers could influence their treatment in the ED or even the asylum process even though all participants were informed in advance that the study would have no influence on their treatment or asylum process.

Another important limitation is the bias due to the German language of the questionnaires. The interviewer decided in case of little or no understanding to involve a translator either in person or mostly by phone. The same was used to obtain consent.

The difference between the use of PC and ED in the home country and Switzerland does not necessarily represent a difference in health-seeking behavior but might be due to different structures with different availabilities in the national HCS.

We did not control for big differences such as education and work status and this may act as a confounder for the measured outcomes in this study.

Despite these limitations, we believe the data help to shed important light on the experiences of AS patients in the HCS. The findings are relevant for all stakeholders involved in clinical care and health care policy and can encourage them to develop and implement new strategies to fill the demonstrated gaps in health care knowledge and improve quality of care.

## 6. Conclusions

Disparities in knowledge of the HCS in the reception country, language barriers, and the perceived level of urgency of medical care seem to be the main reasons for AS to seek care in ED for low-acuity medical issues. In both groups, the decision to present to the ED was influenced by the unlimited access over 24 h, expectation of better treatment in the ED and the perceived level of urgency. 

Measures to increase health literacy and provision of easily accessible primary care could improve quality of care and reduce the usage of EDs as primary care providers to AS. Implementation and usage of a professional interpreting service will relieve family and friends from this role and might provide better and equal care.

## Figures and Tables

**Table 1:** Study population demographic characteristics.

	Asylum Seekers (*n* = 106)	Controls (*n* = 68)	*p*
Median age (IQR)	25 (21–37)	30 (25–41)	0.034
Gender (males), *n* (%)	68 (64.2)	35 (51.5)	0.097
Work status, *n* (%)			
Employed/self-employed	18 (17)	54 (79.4)	
Student	14 (13.2)	9 (13.2)	
Housewife/man	7 (6.6)	2 (2.9)	
Unemployed	67 (63.2)	1 (1.5)	
Retired	0 (0.0)	2 (2.9)	
Region of origin, *n* (%)			
Switzerland		68 (100)	
Eastern Africa	43 (40.6)		
Northern Africa	6 (5.7)		
Southern Asia	18 (17)		
Southern Europe	1 (0.9)		
Western Africa	5 (4.7)		
Western Asia	32 (30.2)		
Missing	1 (0.9)		

**Table 2:** Reasons why patients did not try to or use GP before presenting to the emergency department. (Patients with direct ED consultation N-AS: 67; N-CG: 46; multiple answers possible.)

Reasons	Study Group *n* (%)	Control Group *n* (%)
No GP	20 (29.9)	6 (13)
Calling medical helpline	1 (1.5)	5 (10.9)
Consultation outside visiting hours	17 (25.4)	15 (32.6)
Previous bad experience	13 (19.4)	5 (10.9)
Expected better treatment in ED	13 (19.4)	7 (15.2)
Highly urgent problem	15 (22.4)	10 (21.7)
Missing/Not stated	2 (3)	5 (10.9)

## References

[1] UNHCR Global Trends Forced Displacement in 2016. http://www.unhcr.org/5943e8a34.pdf.

[2] SEM S.f.M. Migrationsbericht 2016. https://www.sem.admin.ch/dam/data/sem/publiservice/berichte/migration/migrationsbericht-2016-d.pdf.

[3] Müller M., Klingberg K., Srivastava D., Exadaktylos A.K. (2016). Consultations by asylum seekers: Recent trends in the emergency department of a Swiss university hospital. PLoS ONE.

[4] Ruud S.E., Aga R., Natvig B., Hjortdahl P. (2015). Use of emergency care services by immigrants - a survey of walk-in patients who attended the Oslo Accident and Emergency Outpatient Clinic. BMC Emerg. Med..

[5] Norredam M., Krasnik A., Sorensen T.M., Keiding N., Michaelsen J.J., Nielsen A.S. (2004). Emergency room utilization in Copenhagen: A comparison of immigrant groups and Danish-born nationals. Scand. J. Public Health.

[6] Norredam M., Mygind A., Nielsen A.S., Bagger J., Krasnik A. (2007). Motivation and relevance of emergency room visits among immigrants and patients of Danish origin. Eur. J. Public Health.

[7] Petersen L.A., Burstin H.R., O’Neil A.C., Orav E.J., Brennan T.A. (1998). Nonurgent emergency department visits: The effect of having a regular doctor. Med. Care.

[8] Ballotari P., D’Angelo S., Bonvicini L., Broccoli S., Caranci N., Candela S., Giorgi Rossi P. (2013). Effects of immigrant status on Emergency Room (ER) utilisation by children under age one: A population-based study in the province of Reggio Emilia (Italy). BMC Health Serv. Res..

[9] Pines J.M., Hilton J.A., Weber E.J., Alkemade A.J., Al Shabanah H., Anderson P.D., Bernhard M., Bertini A., Gries A., Ferrandiz S. (2011). International perspectives on emergency department crowding. Acad. Emerg. Med..

[10] Lin M.P., Baker O., Richardson L.D., Schuur J.D. (2018). Trends in Emergency Department Visits and Admission Rates Among US Acute Care Hospitals. JAMA Intern. Med..

[11] Lowthian J.A., Curtis A.J., Cameron P.A., Stoelwinder J.U., Cooke M.W., McNeil J.J. (2011). Systematic review of trends in emergency department attendances: An Australian perspective. Emerg. Med. J. EMJ.

[12] Jayaprakash N., O’Sullivan R., Bey T., Ahmed S.S., Lotfipour S. (2009). Crowding and delivery of healthcare in emergency departments: The European perspective. West. J. Emerg. Med..

[13] Pei Y.V., Xiao F. (2011). Emergency medicine in China: Present and future. World J. Emerg. Med..

[14] Unwin M., Kinsman L., Rigby S. (2016). Why are we waiting? Patients’ perspectives for accessing emergency department services with non-urgent complaints. Int. Emerg. Nurs..

[15] Uscher-Pines L., Pines J., Kellermann A., Gillen E., Mehrotra A. (2013). Emergency department visits for nonurgent conditions: Systematic literature review. Am. J. Manag. Care.

[16] Keidar O., Jegerlehner S.N., Ziegenhorn S., Brown A.D., Muller M., Exadaktylos A.K., Srivastava D.S. (2018). Emergency Department Discharge Outcome and Psychiatric Consultation in North African Patients. Int. J. Environ. Res. Public Health.

[17] Kraaijvanger N., Rijpsma D., Willink L., Lucassen P., Leeuwen H., Edwards M. (2017). Why patients self-refer to the Emergency Department: A qualitative interview study. J. Eval. Clin. Pract..

[18] Khan Y., Glazier R.H., Moineddin R., Schull M.J. (2011). A population-based study of the association between socioeconomic status and emergency department utilization in Ontario, Canada. Acad. Emerg. Med. Off. J. Soc. Acad. Emerg. Med..

[19] Kraaijvanger N., van Leeuwen H., Rijpsma D., Edwards M. (2016). Motives for self-referral to the emergency department: A systematic review of the literature. BMC Health Serv. Res..

[20] Exadaktylos A.K., Hautz W. (2015). Emergency Medicine in Switzerland. Icu Manag. Pract..

[21] Von Elm E., Altman D.G., Egger M., Pocock S.J., Gøtzsche P.C., Vandenbroucke J.P. (2007). The Strengthening the Reporting of Observational Studies in Epidemiology (STROBE) statement: Guidelines for reporting observational studies. Lancet.

[22] Rutschmann O.T., Sieber R.S., Hugli O.W. (2009). Recommandations de la SSMUS pour le triage dans les services d’urgences hospitaliers en Suisse. Bull. Des. Méd. Suisses.

[23] The World Medical Association (2013). World Medical Association Declaration of Helsinki: Ethical principles for medical research involving human subjects. JAMA.

[24] Arie S. (2019). Countries must do more to help migrants access healthcare, says WHO. BMJ.

[25] Robertshaw L., Dhesi S., Jones L.L. (2017). Challenges and facilitators for health professionals providing primary healthcare for refugees and asylum seekers in high-income countries: A systematic review and thematic synthesis of qualitative research. BMJ Open.

[26] Park J., Johantgen M.E. (2017). A Cross-Cultural Comparison of Symptom Reporting and Symptom Clusters in Heart Failure. J. Transcult. Nurs. Off. J. Transcult. Nurs. Soc..

[27] Bischoff A., Bovier P.A., Rrustemi I., Gariazzo F., Eytan A., Loutan L. (2003). Language barriers between nurses and asylum seekers: Their impact on symptom reporting and referral. Soc. Sci. Med..

[28] Karliner L.S., Jacobs E.A., Chen A.H., Mutha S. (2007). Do professional interpreters improve clinical care for patients with limited English proficiency? A systematic review of the literature. Health Serv. Res..

[29] Shandro J., Chisolm-Straker M., Duber H.C., Findlay S.L., Munoz J., Schmitz G., Stanzer M., Stoklosa H., Wiener D.E., Wingkun N. (2016). Human Trafficking: A Guide to Identification and Approach for the Emergency Physician. Ann. Emerg Med..

[30] Klukowska-Roetzler J., Eracleous M., Muller M., SNivastava D.S., Krummrey G., Keidar O., Exadaktylos A.K. (2018). Increased Urgent Care Centre Visits by Southeast European Migrants: A Retrospective, Controlled Trial from Switzerland. Int. J. Environ. Res. Public Health.

